# A Pilot Study on Single-Cell Raman Spectroscopy Combined with Machine Learning for Phenotypic Characterization of *Staphylococcus aureus*

**DOI:** 10.3390/microorganisms13061333

**Published:** 2025-06-08

**Authors:** Li Liu, Junjing Xue, Yang Song, Taijie Zhan, Yang Liu, Xiaohui Song, Li Mei, Duochun Wang, Yu Vincent Fu, Qiang Wei

**Affiliations:** 1 National Pathogen Resource Center, Chinese Center for Disease Control and Prevention, Beijing 102206, China; liuli022400@163.com (L.L.); lyioiyl@163.com (Y.L.); xhsong77@163.com (X.S.); 2 China General Microbiological Culture Collection Center (CGMCC), State Key Laboratory of Microbial Diversity and Innovative Utilization, Institute of Microbiology, Chinese Academy of Sciences, Beijing 100101, China; 17860693070@163.com; 3 Key Laboratory of Surveillance and Early-Warning on Infectious Disease, National Key Laboratory of Intelligent Tracking and Forecasting for Infectious Diseases, Chinese Center for Disease Control and Prevention, Beijing 102206, China; songyang@chinacdc.cn; 4 School of Health Science and Engineering, University of Shanghai for Science and Technology, Shanghai 200093, China; taijiezhan@163.com; 5 Tuberculosis Prevention and Control Institute, Beijing Center for Disease Control and Prevention, Beijing 100013, China; ml980821@163.com; 6 National Institute for Communicable Disease Control and Prevention, Chinese Center for Disease Control and Prevention, Beijing 102206, China; wangduochun@icdc.cn

**Keywords:** Raman spectroscopy, machine learning, *Staphylococcus aureus*, phenotypic characteristics, rapid detection

## Abstract

Rapid and accurate identification of pathogenic bacteria phenotypic traits, including virulence, drug resistance, and metabolic activity, is essential for clinical diagnosis and infectious disease control. Traditional methods are time-consuming, highlighting the need for more efficient approaches. This study develops a single-cell Raman spectroscopy approach to detect multiple phenotypic traits of *Staphylococcus aureus* (*S. aureus*) as a proof of concept. We constructed a single-cell Raman spectral database encompassing 6240 spectra from 10 strains of *S. aureus* with diverse phenotypic traits and developed a convolutional neural network (CNN) to predict these phenotypes from the Raman spectra. The CNN model achieved 93.90%, 98.73%, and 98.66% accuracy in identifying enterotoxin-producing strains, methicillin-resistant *S. aureus* (MRSA), and growth stages, respectively. Characteristic Raman peaks for enterotoxin producers mainly appeared at 781, 939, 1161, 1337, 1451, and 1524 cm^−1^, whereas MRSA primarily exhibited peaks at 723, 780, 939, 1095, 1162, 1340, 1451, 1523, and 1660 cm^−1^. During culture, nucleic acid-related peaks weakened, lipid peaks increased, and protein peaks initially increased and subsequently decreased. This integration of Raman spectroscopy and machine learning demonstrates considerable potential for rapid bacterial phenotyping. Future research should expand to a wider range of bacterial species and phenotypes to enhance the diagnosis, prevention, and management of infectious diseases.

## 1. Introduction

*Staphylococcus aureus* (*S. aureus*), a prevalent pathogen, easily colonizes within human body and causes various diseases [1]. It is one of the major contributors to bacterial food poisoning, and its production of enterotoxins can lead to acute gastroenteritis or even death [2]. To date, 24 types of staphylococcal enterotoxins and enterotoxoids have been identified, with five being the most common: SEA, SEB, SEC, SED, and SEE [3]. These toxins are implicated in approximately 95% of *S. aureus*-related food poisoning cases, posing a significant global public health concern [4]. In recent years, the abuse of antibiotics has led to the continuous emergence of drug resistance in *S. aureus*. Among them, methicillin-resistant *S. aureus* (MRSA) poses the greatest threat and is known as the “superbug” [5]. In the 2024 list of priority pathogens of the World Health Organization, MRSA is classified as a pathogen of high priority. Compared with methicillin-susceptible *S. aureus* (MSSA), MRSA is closely associated with a relatively high annual mortality rate globally [6,7]. In 2019 alone, MRSA accounted for over 100,000 deaths worldwide due to its resistance to antibiotics [8]. As the leading cause of infection-related deaths, MRSA also ranks as the most frequent pathogen in secondary co-infections, imposing a heavy economic burden on healthcare systems and negatively impacting the global economy [9,10,11,12].

During the growth process, bacteria need to go through four stages: the lag phase, the log phase, the stationary phase, and the decline phase [13]. Strains at different growth stages exhibit significant differences in metabolic activity, and the degrees and manifestations of the harm they cause also vary [13,14]. Therefore, by detecting the growth stage of a strain, its potential harm level can be inferred. Meanwhile, as a key influencing factor, the cultivation duration will change the internal structure and molecular vibration modes of the strain, which may in turn lead to changes in the positions, intensities, and relative proportions of the characteristic peaks in the Raman spectrum [15]. This indicates that when constructing a comprehensive Raman spectrum database of pathogenic bacteria, the influence of the cultivation time of strains on the spectral characteristics needs to be fully considered.

Currently, the identification of *S. aureus* enterotoxin mainly relies on the Enzyme-Linked Immunosorbent Assay (ELISA) experiments [16]; the detection of antibiotic susceptibility mainly refers to methods such as the disk diffusion method and the broth microdilution method recommended by the guidelines of the European Committee on Antimicrobial Susceptibility Testing (EUCAST) and the Clinical and Laboratory Standards Institute (CLSI) [17]. The most traditional method for detecting the activity of pathogenic bacteria is the plate count method based on growth ability, but it cannot distinguish between metabolically dormant cells and dead cells [18]. As a molecular diagnostic technique, Polymerase Chain Reaction (PCR) has the ability to detect pathogenic bacteria and has the advantages of being rapid and sensitive. However, its detection scope is limited to the verification of the presence of genes, and it cannot detect the gene expression status, which needs to be confirmed by combining with phenotypic verification experiments [19,20]. Although real-time fluorescence quantitative PCR can distinguish between live and dead bacteria, it requires the combination of fluorescent labeling, and the cost is relatively high [21]. Advanced technologies such as flow cytometry and mass spectrometry are costly, have strict requirements for sample pretreatment, and require cumbersome steps such as extraction and purification. Moreover, they have disadvantages such as being unable to conduct non-destructive detection at the single-cell level.

In recent years, Raman technology has gradually been applied in the field of pathogenic microorganisms. Raman technology can provide unique “fingerprint” information for each bacterial cell [22]. The fingerprint region of the Raman spectrum can characterize the internal structure and biomolecules of single cells, such as biological macromolecules like nucleic acids, proteins, carbohydrates, and lipids [23]. In addition, the use of Raman spectroscopy does not require pretreatment, and it only takes a few seconds to detect pathogenic bacteria. However, it is difficult to distinguish the types and phenotypic characteristics of pathogenic bacteria solely through Raman spectroscopy. This is because the Raman spectrum is easily affected by background noise, and there are many Raman spectral bands, making it difficult to distinguish them with the naked eye [24]. Therefore, it is necessary to use chemometrics in combination with Raman spectroscopy to achieve rapid and accurate identification of the types and phenotypes of pathogenic bacteria.

As a statistical tool, machine learning can extract features from raw data to solve problems. It mainly includes two types: unsupervised learning and supervised learning [25]. Principal component analysis (PCA), as an unsupervised learning method, can classify the dataset according to the similarity of the input data of unknown types and is a commonly used method for dimensionality reduction [26,27,28]. As a supervised learning method, convolutional neural networks (CNNs) offer considerable advantages for processing large-scale datasets and exhibit strong performance in identifying pathogenic bacteria based on Raman spectroscopy [23,29,30]. Therefore, in this study, *S. aureus* was used as a proof of concept. Raman spectroscopy combined with deep learning was employed to identify *S. aureus* with different phenotypes, thus providing a new method for the single-cell phenotypic detection of pathogenic bacteria.

## 2. Materials and Methods

### 2.1. Experimental Strains

This experiment encompassed ten strains of *S. aureus* demonstrating distinct phenotypic characteristics. Seven *S. aureus* strains were obtained from the National Pathogen Resource Center (NPRC) of the Chinese Center for Disease Control and Prevention (CDC). Additionally, two strains were acquired from the Sichuan Provincial Center for Disease Control and Prevention, and one strain from the Guangdong Provincial Microbial Species Preservation Center. All strains were identified and subjected to second-generation sequencing (Supplementary Materials Table S1).

### 2.2. Enterotoxin Identification Experiment

The detection experiment of *S. aureus* enterotoxin was strictly carried out in accordance with the “Microbiological examination of food-Examination of *S. aureus*” (GB4789.10-2016) [31]. The colonies on the nutrient agar plate were eluted with 5 mL of normal saline and transferred to 60 mL of enterotoxin-producing medium (Qingdao Hi-tech Industrial Park Hope Biotechnology Co., Ltd., Qingdao, China). They were cultured with shaking at 36 °C for 48 h, inactivated by heating at 100 °C for 10 min, and then centrifuged (8000× *g*, 2 min). A total of 100 µL of the diluted supernatant was taken as the sample, and the experiment was conducted according to the instruction manual of the *S. aureus* enterotoxin typing kit produced by Yizhi Technology Co., Ltd. (Shenzhen, China).

Result judgment: When the optical density (OD) value of the negative control is less than 0.2 and that of the positive control is greater than 0.5, the threshold (T) is calculated as the average value of the two negative controls plus 0.2: $T=\frac{NC1+NC2}{2}+0.2$. If the OD value of the sample is greater than or equal to T, the sample is positive; if the OD value of the sample is less than T, the sample is negative.

### 2.3. Susceptibility Testing

The sensitivity of the experimental strains to cefoxitin was evaluated by the Kirby–Bauer disk diffusion method, with the *S. aureus* strain ATCC25923 serving as the control strain. Before the experiment, all strains were sub-cultured on Luria–Bertani (LB) solid plates (Sinopharm Chemical Reagent Co., Ltd, Shanghai, China) and incubated at 37 °C for 18–24 h. The bacterial suspension of the experimental strains was adjusted to the McFarland 0.5 turbidity standard (approximately 1.5 × 10^8^ CFU/mL) with sterile saline and then evenly spread on Mueller–Hinton agar plates (Qingdao Hi-tech Industrial Park Hope Biotechnology Co., Ltd., Qingdao, China) using a sterile cotton swab. The disks containing cefoxitin were placed on the plates, and the plates were inverted and incubated at 37 °C for 16–18 h. The diameter of the inhibition zone was measured to the nearest millimeter using a high-precision measuring tool. The drug susceptibility results were interpreted according to the guidelines of the CLSI [32].

### 2.4. Measurement of Experimental Strain Growth Curve

The strains stored at −80 °C were inoculated onto LB solid medium. After incubation at 37 °C for 18–24 h, single colonies were selected and cultured in 5 mL shake tubes at 37 °C for an additional 18–24 h. Subsequently, when the bacterial solution was diluted to McFarland turbidity 0.5, it was transferred to another 5 mL of LB medium at a 1:100 dilution for further culture. The optical density at 600 nm (OD_600_) was measured at 0, 1, 2, 3, 4, 6, 8, 10, 12, 16, 20, and 24 h, to determine the growth curves of *S. aureus* strains.

### 2.5. Single-Cell Raman Spectrum Acquisition

All experimental strains were cultured under identical preparation conditions in LB medium. Moreover, three independent batches of bacterial solutions were collected to cover a certain degree of variability within the dataset. Phenotypic strains were cultured for 18–24 h. *S. aureus* samples were taken at different growth phases for analysis: lag phase (1.5 h), log phase (6 h), and stationary phase (14 h). Sample preparation: the bacterial solution, cultured for 18–24 h, was centrifuged at 8000× *g* for 2 min. Three ddH_2_O washes were performed (8000× *g*, 2 min) to remove the medium. The experimental bacterial solution was then diluted to 10^5^ bacteria/mL using 0.85% NaCl solution. Raman spectra were measured using laser tweezers Raman spectroscopy (LTRS) operating at 785 nm [33]. Polystyrene spheres with a diameter of 10 µm were used for spectral calibration at peaks of 620.9, 1001.4, and 1602.3 cm^−1^. The collection time for each bacterial spectrum was set at 25 s to enhance the signal-to-noise ratio and ensure consistency across strain measurements. The Raman spectra were organized into a training set comprising three different experimental batches and an independent test set from an entirely separate batch.

### 2.6. Pretreatment and Modeling of Single-Cell Raman Spectra

In this study, the self-developed Ramanpro Raman spectroscopy analysis software package, which is developed based on the R language (Version 3.4.3) architecture, was used to perform standardized preprocessing operations on the Raman spectra of *S. aureus*. The specific preprocessing procedures are as follows: Firstly, the background was removed; then, the Savitzky–Golay filtering algorithm was applied to smooth the Raman spectra; subsequently, the baseline was corrected using the asymmetric least squares method; thereafter, through vector normalization processing, the spectral signals were adjusted to a unified scale range; and data quality control was carried out by means of the K-means clustering method.

In addition, in this study, the R language (Version 3.4.3) and RStudio (Version 3.4.3) were employed. After the above-mentioned preprocessing steps were completed, dimensionality reduction was performed on the spectral fingerprint region (600–1800 cm^−1^) through PCA. A CNN was used to perform 10-fold cross-validation on the training set to construct a phenotypic identification model for *S. aureus*. Meanwhile, independent test sets were used to evaluate the different constructed phenotypic identification models for *S. aureus*. Moreover, the receiver operating characteristic (ROC) curve and the area under curve (AUC) were utilized to quantitatively evaluate the accuracy of the model in the phenotypic identification of *S. aureus*.

## 3. Result

### 3.1. Phenotypic Characteristics of the Experimental Strains

**Figure 1:**
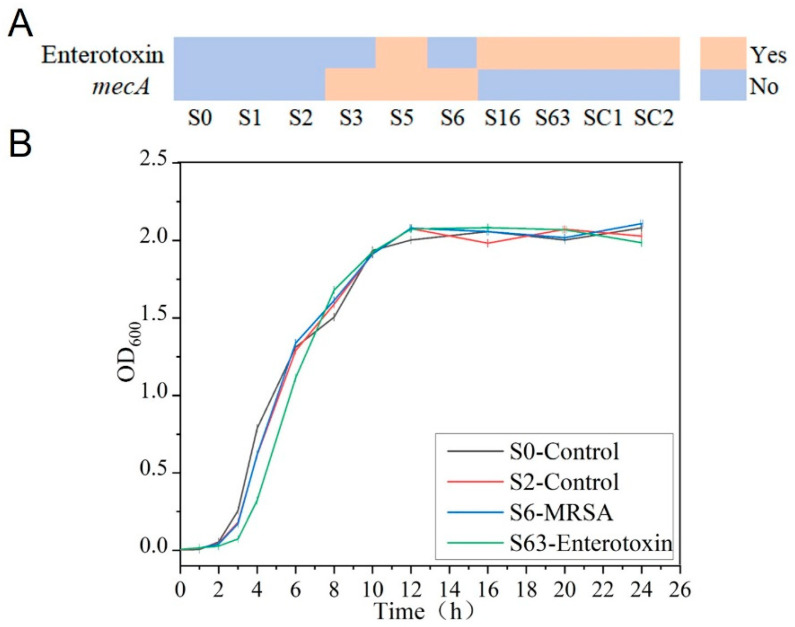
Phenotypic characteristics of the experimental strains. (**A**) Expression of enterotoxin and *mecA*. (**B**) Growth curve of *S. aureus*.

In this study, the expression status of enterotoxin-related genes was determined by ELISA. The results showed that strains S0, S1, S2, S3, and S6 did not produce enterotoxin, whereas strains S5, S16, S63, SC1, and SC2 were capable of enterotoxin production (Figure 1A). In addition, in accordance with the research protocol and CLSI guidelines, the Kirby–Bauer disk diffusion method was used to assess the susceptibility of the experimental strains to cefoxitin. The experimental results clearly indicated that strains S3, S5, and S6 exhibited resistance to cefoxitin, whereas the remaining seven *S. aureus* strains were sensitive (Figure 1A). These results strongly confirmed the presence of the *mecA* in strains S3, S5, and S6, identifying them as MRSA, while the remaining strains were classified as MSSA.

To further investigate the growth patterns of *S. aureus*, the strains were continuously cultured for 24 h, and the OD_600_ values of the bacterial suspensions were measured at regular intervals throughout the culture process to plot growth curves. By plotting culture time on the *x*-axis and OD_600_ values on the *y*-axis, it was found that the growth trends of the different *S. aureus* strains were highly similar. According to the growth characteristics of the pathogens, during the initial 0–3rd h of culture, all strains were in the lag phase with slow growth. Between the 3rd and 11th h, the OD_600_ values exhibited an exponential increase, indicating that the strains were in the log phase with rapid growth. From the 11th to 24th h, the OD_600_ values plateaued around 2.0 with minimal fluctuations, signifying that the strains had entered the stationary phase.

### 3.2. Construction of Raman Spectral Database of S. aureus

**Figure 2:**
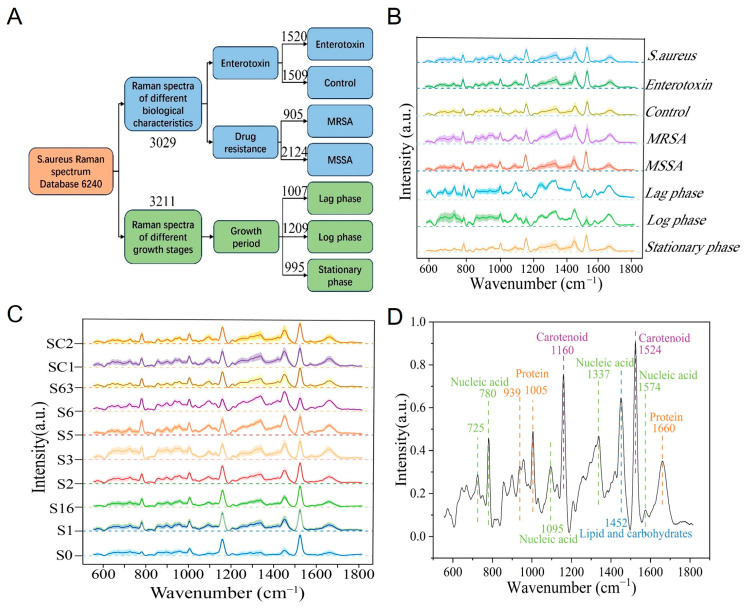
Raman spectral database of *S. aureus*. (**A**) Composition of the *S. aureus* Raman spectral database. (**B**) Average Raman spectra of *S. aureus* with different phenotypes. Solid line represents the average spectrum; shading indicates standard deviation. (**C**) Raman spectra of various *S. aureus* strains. Solid line: average spectrum; shaded area: standard deviation. (**D**) Characteristic Raman peak positions of *S. aureus*.

A total of 6240 Raman spectra were collected in this study, comprising 3029 spectra from 10 *S. aureus* strains to compare biological characteristics across strains, and 3211 spectra from 4 *S. aureus* strains at different growth stages to characterize the metabolic activities representative of each growth period. Of the 3029 spectra for biological characterization, 1520 spectra were collected from five strains characterized as enterotoxin-producing and 1509 spectra from five non-enterotoxin-producing strains of *S. aureus*. Within these 3029 spectra, 905 were collected from three MRSA strains, and 2124 from the seven MSSA strains. Raman spectra at different growth phases comprise 1007 lag phase spectra, 1209 log phase spectra, and 995 stationary phase spectra (Figure 2A).

Through meticulous observation and detailed analysis of Raman spectra, it was found that the Raman spectra of different *S. aureus* strains exhibited a high degree of similarity in their overall morphology, with only subtle differences in the intensity distribution of characteristic peaks. Further comparative analysis of strains with distinct phenotypes revealed that the Raman spectral profiles of enterotoxin-producing and non-enterotoxin-producing strains, as well as MRSA and MSSA, were highly similar, with no substantial differences in the positions and shapes of characteristic peaks. However, the spectra of strains at the lag, log, and stationary phases exhibited distinct intensity variations (Figure 2B,C).

Considering the notable limitations of microscopic identification methods based on visual assessment, such as high subjectivity and low analytical efficiency, this study employed chemometric techniques for spectral feature extraction and identification to enable precise analysis and identification of different phenotypes of *S. aureus*. Chemometric analysis demonstrated that the characteristic Raman peak positions of *S. aureus* were mainly concentrated in the range of 700–1700 cm^−1^, corresponding to the characteristic peaks of nucleic acids, proteins, and lipids (Figure 2D and Table 1).

### 3.3. Raman Spectral Identification and Analysis of Enterotoxin-Producing and Non-Enterotoxin-Producing S. aureus

**Figure 3:**
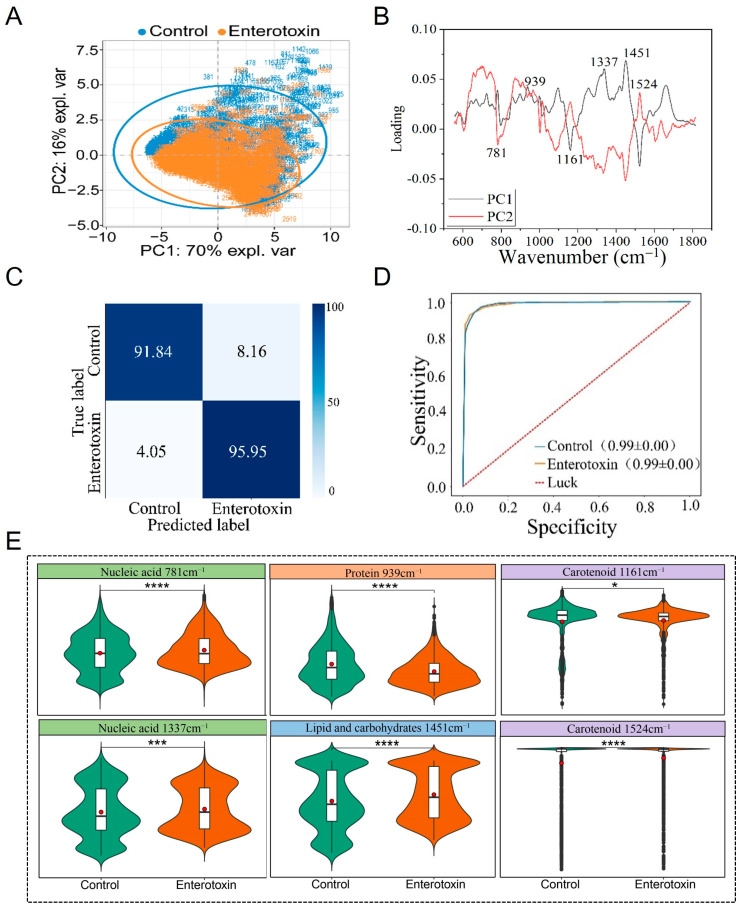
Raman spectra comparison of enterotoxin-producing and non-enterotoxin-producing *S. aureus*. (**A**) PCA. (**B**) PCA loading analysis. (**C**) Prediction of enterotoxin-producing and non-enterotoxin-producing *S. aureus* by the CNN model. (**D**) ROC curve for CNN model evaluation. (**E**) Violin plot of characteristic Raman peak positions. * *p* < 0.05, *** *p* < 0.001, **** *p* < 0.0001.

PCA demonstrated that enterotoxin-producing and non-enterotoxin-producing *S. aureus* could not be distinguished (Figure 3A). PCA loading analysis revealed that the key differences were located at Raman peaks (cm^−1^) 781 (nucleic acid) [34,35], 939 (protein) [36,37], 1161 (carotenoid) [40], 1337 (nucleic acid) [34], 1451 (lipid and carbohydrate) [41,42], and 1524 (carotenoid) [40] (Table 1), all of which were statistically significant (*p* < 0.05) (Figure 3B,E). The characteristics corresponding to these peak positions may provide potential references for phenotypic identification, though it should be noted that their relevance is not absolutely definitive.

The CNN model with the optimal performance following multiple rounds of training was selected to predict an independently collected Raman spectroscopy test set that had not been used during model training. The results showed that the model achieved an accuracy of 95.95% for identifying enterotoxin-producing *S. aureus*, 91.84% for identifying non-enterotoxin-producing *S. aureus*, and an overall accuracy of 93.90% (Figure 3C). Using ten-fold cross-validation, the recall and precision of the Raman spectra for the two types of strains during the training process were systematically evaluated, and the ROC curve was subsequently plotted. The study demonstrated that the AUC reached as high as 0.99, indicating the model’s excellent capability for identifying Raman spectra of enterotoxin-producing and non-enterotoxin-producing *S. aureus* (Figure 3D).

### 3.4. Raman Spectral Identification and Analysis of MRSA and MSSA Strains

**Figure 4:**
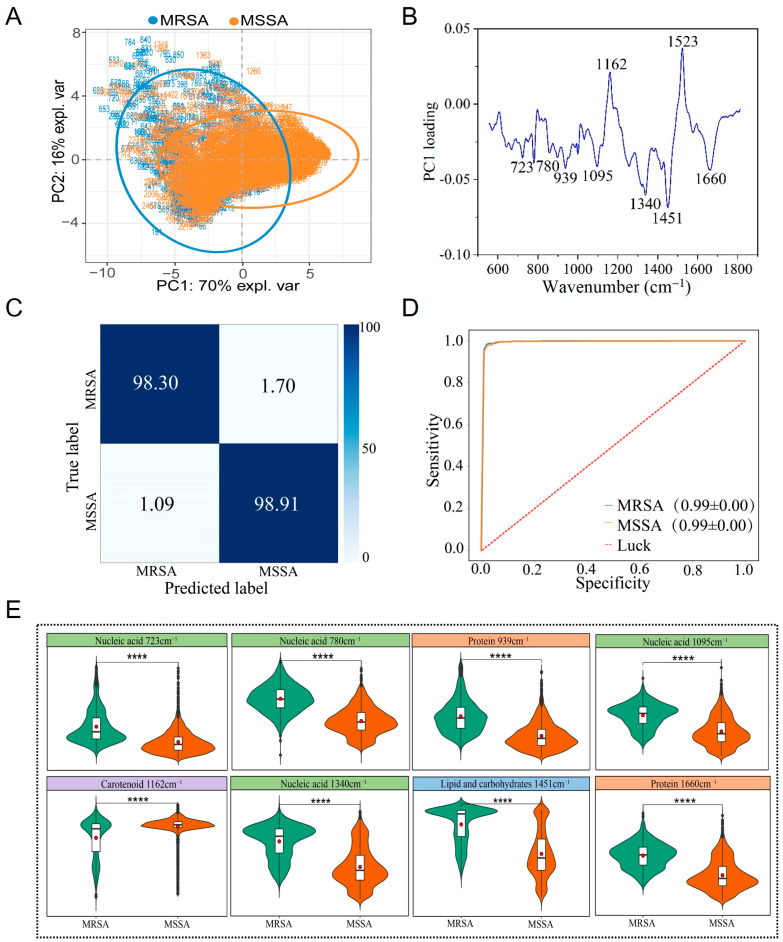
Raman spectra of MRSA and MSSA strains. (**A**) PCA. (**B**) PCA loading analysis. (**C**) Prediction of MRSA and MSSA by the CNN model. (**D**) ROC curve for evaluating the CNN model. (**E**) Violin plot of characteristic Raman peak positions. **** *p* < 0.0001.

**Figure 5:**
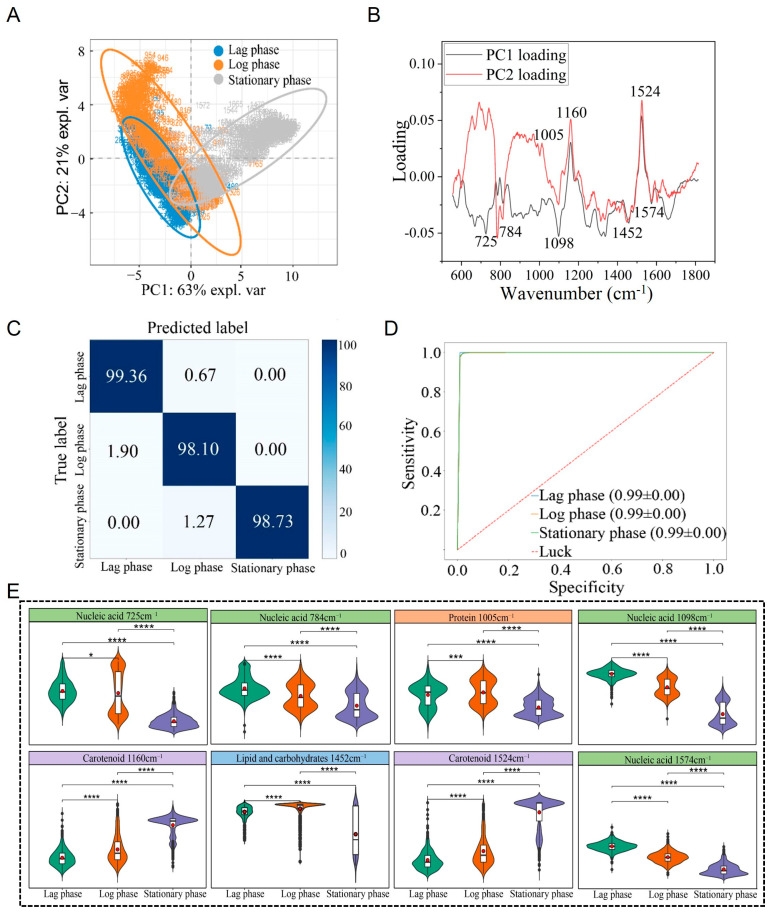
Raman spectrum identification and analysis of *S. aureus* across different growth stages. (**A**) PCA. (**B**) PCA loading analysis. (**C**) Prediction of *S. aureus* in different periods by the CNN model. (**D**) ROC curves evaluated against the CNN model. (**E**) Violin plot of characteristic Raman peak positions. * *p* < 0.05, *** *p* < 0.001, **** *p* < 0.0001.

After completing the classification of MRSA and MSSA, this study conducted an in-depth analysis for precise identification. The results of PCA revealed that the Raman spectra of MRSA and MSSA exhibited distinct clustering characteristics, although some overlap was observed between the two groups (Figure 4A). Analysis of the PC1 dimension of the Raman spectra showed that characteristic identification peaks were mainly concentrated in the following regions: peaks associated with nucleic acids (723, 780, 1095, 1340 cm^−1^) [34,35,36]; peaks associated with proteins (939, 1660 cm^−1^) [35,36,37,47]; peaks associated with carotenoid (1162, 1523 cm^−1^) [40]; and peaks with mixed characteristics of lipids and carbohydrates (1451 cm^−1^) [41,42]. One-way analysis of variance demonstrated that the intensity differences in these characteristic peaks were statistically significant (*p* < 0.05) (Figure 5B,E).

To achieve rapid and accurate identification of MRSA and MSSA, this study applied a CNN for further classification. Using the established CNN model, predictions were made on an independent Raman spectroscopy test set, and the results showed that the classification model achieved excellent performance. The identification accuracy for MRSA reached 98.30%, the classification accuracy for MSSA was 98.91%, and the overall classification accuracy was as high as 98.73% (Figure 4C). The model’s performance was evaluated using the ROC curve, and the AUC value reached 0.99, further verifying the model’s excellent sensitivity and specificity (Figure 4D).

### 3.5. Raman Spectral Identification and Analysis of S. aureus at Different Growth Stages

The potential hazards posed by pathogenic bacteria can vary depending on their growth stages. Additionally, the cultivation time of *S. aureus* can influence its Raman spectra. To conduct an in-depth analysis of the differences in the Raman spectra of *S. aureus* at different growth stages, this study initially selected standard strains S0 and S2. These two standard strains neither produce enterotoxin nor exhibit methicillin resistance. By collecting their Raman spectra at different growth stages, the aim was to achieve rapid and accurate detection of *S. aureus*.

Following clustering analysis via PCA, it was found that the Raman spectra of *S. aureus* during the lag phase, log phase, and stationary phase could be clearly separated into distinct clusters (Figure 5A). PCA loading analysis identified key Raman peaks at 725 cm^−1^ (nucleic acid) [34], 784 cm^−1^ (nucleic acid) [34,35], 1005 cm^−1^ (protein) [38,39], 1098 cm^−1^ (nucleic acid) [36], 1160 cm^−1^ (carotenoid) [40], 1452 cm^−1^ (lipid and carbohydrate) [41,42], 1524 cm^−1^ (carotenoid), and 1574 cm^−1^ (nucleic acid) [34,45,46] as the primary contributors to these stage-specific differences (Table 1). These differences were statistically significant (*p* < 0.05) (Figure 5B,E).

To further facilitate differentiation and identification of *S. aureus* at different growth stages, this study applied a CNN for analysis. Using the established CNN model, predictions were made for the Raman spectra of *S. aureus* at the independent lag, log, and stationary phases, achieving an average identification accuracy of 98.66%. Among 524 Raman spectra, 517 were correctly classified. Specifically, the identification accuracy for the lag phase was 99.36%, with only 0.64% misclassified as the logarithmic phase; the accuracy for the logarithmic phase was 98.10%, with 1.90% misclassified as the lag phase; and the accuracy for the stationary phase was 98.73%, with 1.27% misclassified as the logarithmic phase (Figure 5C). The model’s performance was evaluated using the ROC curve, and the AUC value reached 0.99, indicating strong sensitivity and specificity (Figure 5D).

To further explore whether enterotoxin production and methicillin resistance would affect the CNN model’s identification of *S. aureus* growth stages, this study also included the enterotoxin-producing strain S63 and the MRSA S6. Their Raman spectra at different growth stages were collected to validate the CNN model for growth stage identification. The average identification accuracies for S63 and S6 were 94.60% and 94.03%, respectively (Supplementary Materials Figure S1A,B). Although these accuracies were slightly lower than those observed for the independent test sets of standard strains, this did not compromise the overall identification performance.

### 3.6. Identification of Different Phenotypes of S. aureus Cultured for Different Durations

**Figure 6:**
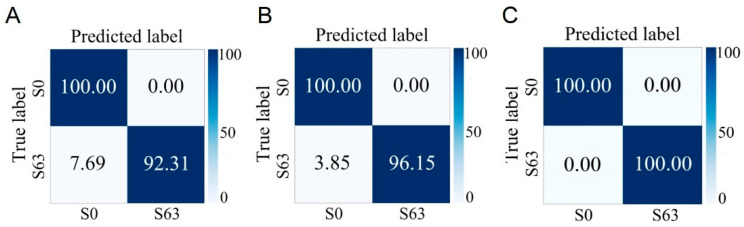
Prediction of non-enterotoxin-producing *S. aureus* (S0) and enterotoxin-producing *S. aureus* (S63) cultured at different growth stages by the CNN model. (**A**) Lag phase. (**B**) Logarithmic phase. (**C**) Stationary phase.

**Figure 7:**
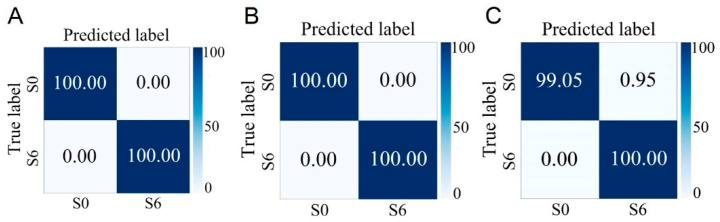
Prediction of MSSA (S0) and MRSA (S6) cultured at different growth stages by the CNN model. (**A**) Lag phase. (**B**) Logarithmic phase. (**C**) Stationary phase.

Based on the above series of analysis and identification results, it can be seen that the Raman spectra of *S. aureus* at different cultivation times are different, and this difference may have an impact on the rapid and accurate identification of different phenotypes of *S. aureus*. In addition, shortening the cultivation time of strains to improve detection speed is of great significance. To this end, in this study, the Raman spectrum data collected previously at different growth stages were classified according to two criteria: whether enterotoxin is produced and whether it is MRSA. The research results show that regardless of whether the strain is in the lag phase, log phase, or stationary phase, as long as the cultivation time is the same, it is possible to accurately distinguish between enterotoxin-producing and non-enterotoxin-producing *S. aureus*, as well as between MRSA and MSSA. The average accuracies of using strains in the lag phase to identify strains with different phenotypes reach 97.45% and 100.00%, which greatly shortens the detection time (Figure 6 and Figure 7).

## 4. Discussion

Globally, public health issues caused by *S. aureus* remain a significant concern. The enterotoxins produced by *S. aureus* and MRSA are key factors contributing to foodborne diseases and clinical infections. Therefore, the ability to rapidly and accurately detect the phenotypic characteristics of *S. aureus* is of great significance for preventing and controlling the transmission and outbreaks of related diseases. This study demonstrates that Raman spectroscopy combined with machine learning enables rapid and accurate simultaneous identification of enterotoxin-producing and non-enterotoxin-producing *S. aureus*, MRSA, MSSA, and *S. aureus* at different growth stages at the single-cell level. Compared to traditional detection methods, this approach does not require extended cultivation of strains. With a cultivation period of just 1.5 h, rapid identification can be achieved, thus significantly enhancing detection efficiency. Moreover, this method overcomes the limitations of traditional techniques that require multiple tests to characterize multiple phenotypic traits, allowing simultaneous identification of several key phenotypic traits. This detection approach offers notable advantages, including reduced time consumption, low cost, and non-destructive analysis. It holds significant promise for applications in areas such as the prevention and control of foodborne diseases and the diagnosis of clinical infections and is expected to become an important technical tool for pathogen detection in the future.

### 4.1. Analysis and Discussion of Enterotoxin-Producing and Non-Enterotoxin-Producing S. aureus

This study is the first to explore the potential of Raman spectroscopy combined with machine learning for distinguishing between enterotoxin-producing and non-enterotoxin-producing *S. aureus*. Notably, this method overcomes the 48 h requirement of the ELISA test for enterotoxin production and the associated complex experimental procedures. The spectral acquisition time is only 25 s, thereby greatly improving detection efficiency. Additionally, Raman spectroscopy can reveal significant changes in substances such as nucleic acids, proteins, and lipids within pathogen cells, facilitating the differentiation of metabolic characteristics among cells within the same taxonomic group [43]. In enterotoxin-producing *S. aureus*, the intensities of peaks representing nucleic acids (781 and 1337 cm^−1^) and proteins (939 cm^−1^) are relatively strong (*p* < 0.05), potentially related to the transcription of enterotoxin genes and the synthesis of enterotoxins actively secreted by the bacteria [48]. Among these, 1337 cm^−1^ corresponds to the vibration mode of the purine ring, consistent with enhanced nucleic acid synthesis and transcriptional activity, whereas the Raman peak at 939 cm^−1^ is attributed to proteins, likely reflecting the accumulation of enterotoxin proteins [34,36]. Furthermore, the intensities of carotenoid-associated peaks (1161 and 1524 cm^−1^) in enterotoxin-producing *S. aureus* are reduced, presumably due to the redirection of energy resources towards virulence factor synthesis. These characteristic Raman peak positions provide a critical basis for the identification of enterotoxin-producing *S. aureus*. It should be noted, however, that these peak positions may be associated with the identification of enterotoxigenic and non-enterotoxigenic *S. aureus* but are not directly linked to the pathogenicity of *S. aureus*.

### 4.2. Analysis and discussion of MRSA and MSSA

There have been some studies that utilize Raman technology to detect drug-resistant bacteria and drug-sensitive bacteria [49,50,51]. Previous studies based on the detection of a single drug-resistant phenotype have employed Raman spectroscopy to distinguish between MRSA and MSSA, achieving an accuracy of 89.0% ± 0.1% [52]. Studies have shown that Raman spectroscopy is a rapid and reliable method for MRSA typing, offering more advantages than pulsed field gel electrophoresis [53]. A deep neural network based on the stacked autoencoder was used to rapidly identify MRSA and MSSA through label-free surface-enhanced Raman scattering technology, with an accuracy rate of 97.66% [54]. However, this technology was not applied to live cell collection. The CNN model developed in this study achieved an identification accuracy of 97.03% for MRSA in an independent test set and demonstrated the ability to detect multiple phenotypes of *S. aureus*.

This study found that, compared to MSSA, the peak intensities at characteristic nucleic acid bands (723 cm^−1^, 780 cm^−1^, 1095 cm^−1^) and the protein band (1660 cm^−1^) in MRSA increased significantly (*p* < 0.05). The enhancement of the peaks at 723 cm^−1^ and 780 cm^−1^ may be associated with the elevated transcriptional activity of the *mecA* gene. Metabolomic studies have indicated that the metabolic differences between MRSA and MSSA primarily involve capsule and cell wall biosynthesis, bacitracin accumulation, and purine/pyrimidine metabolism [55]. The enhancement of the protein peak at 1660 cm^−1^ is likely related to the expression of penicillin-binding protein 2a (PBP2a), encoded by *mecA* [56,57]. Drug-resistant bacteria may enhance their viability by upregulating various stress proteins or drug-resistance-associated enzymes, leading to increased protein abundance [58,59]. Additionally, previous studies have shown that the peak positions at 1160 cm^−1^ and 1523 cm^−1^ are closely associated with the differentiation of MRSA and MSSA, and these characteristic peaks are primarily attributed to the carotenoid staphyloxanthin [40,60]. However, the expression levels of staphyloxanthin vary significantly among different strains, and it is not a marker for drug resistance [40]. In this study, the CNN combined with machine learning was used to identify MRSA and MSSA, with the analysis based on the full spectral information in the fingerprint region rather than solely relying on specific Raman peak positions.

### 4.3. Analysis and Discussion of S. aureus at Different Growth Stages

*S. aureus* exhibits distinct physiological, biochemical, and metabolic characteristics during different growth phases, and the hazards associated with bacteria at different stages also vary. For example, strains in the stationary phase produce a greater amount of toxins [61]. In this study, Raman spectroscopy combined with machine learning was used to identify *S. aureus* at different growth stages, achieving an average accuracy of 98.66%, which is higher than the 91.2% reported for distinguishing three growth stages of *Lactobacillus casei* [62]. For enterotoxin-producing *S. aureus* and MRSA, the method maintained an average accuracy exceeding 94.03%. Although the accuracy slightly decreased, this is likely attributable to minor strain-specific differences without compromising the overall identification performance [63]. Future research can incorporate a greater diversity of strains to enhance model robustness and establish a more comprehensive Raman spectral database for *S. aureus*.

This study found that the Raman spectral characteristics of *S. aureus* during the lag, log, and stationary phases were closely associated with nucleic acids, proteins, and lipids, respectively, mirroring findings observed in *Lactobacillus* [62]. In the lag phase, the intensity of nucleic acid-associated Raman peaks was highest [34,36], suggesting active gene replication and transcription to prepare for subsequent proliferation. The nucleic acid content was relatively high during the lag and log phases but decreased significantly during the stationary phase, likely due to nutrient depletion in the environment [64]. In contrast, lipid-associated Raman peaks were most intense during the stationary phase, potentially reflecting stress-induced lipid accumulation [40]. Protein content peaked during the log phase, possibly related to increased synthesis of adhesion factors, adhesion proteins, and virulence proteins critical for biofilm formation [65,66]. Throughout the growth process, nucleic acid production declined progressively, while protein and lipid synthesis increased in response to environmental stress [67].

### 4.4. Analysis and Discussion on the Phenotypic Identification of S. aureus Cultured for Different Durations

To further expedite the detection process, this study conducted an in-depth analysis of *S. aureus* during the lag, log, and stationary phases, aiming to rapidly and accurately identify and distinguish between enterotoxin-producing and non-enterotoxin-producing strains, as well as between MRSA and MSSA. The results demonstrate that this method can accurately identify the aforementioned strain types. Notably, even when the *S. aureus* used for identification was cultured for only 1.5 h, the method successfully distinguished *S. aureus* strains with different phenotypes. This finding significantly enhances detection efficiency and provides strong support for the rapid screening of pathogenic bacteria. It holds significant implications for the targeted diagnosis of clinical pathogenic bacteria and the prevention and control of infectious diseases. Furthermore, this suggests that the establishment of standardized and commercialized Raman spectral databases for pathogenic bacteria requires incorporating Raman spectra from strains at various growth stages to improve identification accuracy.

In this study, Raman spectroscopy technology was integrated with machine learning, resulting in a significant advancement in the field of *S. aureus* detection. This method enables the rapid and accurate simultaneous identification of multiple phenotypes at the single-cell level, including enterotoxin-producing and non-enterotoxin-producing strains, MRSA, MSSA, and different growth stages. Compared with traditional detection techniques, the efficiency of detection has been significantly improved. Only 1.5 h of strain culturing and 25 s of spectral acquisition are required, achieving an average accuracy exceeding 93.90%. Moreover, this method offers substantial advantages, including parallel analysis of multiple phenotypes, non-destructive sample detection, and low cost, demonstrating substantial application potential in clinical diagnosis, treatment, and the prevention and control of infectious diseases. However, some limitations remain in this study. At present, only the overall differentiation between enterotoxin-producing and non-enterotoxin-producing *S. aureus* strains has been achieved, without precise identification of individual enterotoxin-producing strains. Targeted research should be undertaken in the future to further improve detection accuracy. Furthermore, to continuously enhance the accuracy and universality of pathogenic bacteria detection, future research should systematically expand the sample size, incorporate a wider variety of pathogenic genera and phenotypes, and establish a more comprehensive and enriched Raman spectral database, thereby providing a robust data foundation and technical support for the rapid and accurate detection of pathogenic microorganisms.

## 5. Conclusions

In this study, using *S. aureus* as a proof of concept, a Raman spectroscopy-based platform for multi-phenotypic characterization of pathogens was established, which can be regarded as a “medical examination” tool for pathogens. The platform integrates Raman spectroscopy technology with advanced machine learning techniques to rapidly and accurately determine multiple phenotypic characteristics of *S. aureus* at the single-cell level, including enterotoxigenicity, MRSA, and growth stage classification. Notably, this method eliminates the need for multiple independent experiments, as it can simultaneously determine multiple phenotypic characteristics of *S. aureus* using existing Raman spectral databases and trained models. This approach provides a rapid and accurate methodological reference for clinical pathogen diagnosis and the prevention and control of infectious diseases in related fields. In the future, it will be necessary to further expand the types and quantities of pathogenic bacteria and establish a larger Raman spectral database for pathogens to enhance its applicability.

## Figures and Tables

**Table 1:** Assignment of Raman bands in difference spectra.

Wavenumber (cm^−1^)	Component/Wavenumber (cm^−1^)	Reference
723/725	Adenine ring breathing, nucleic acid (724)	[34]
780/781/784	Uracil-based ring breathing, thymine (780), DNA (783), nucleic acid (785)	[34,35]
939	Protein (936)	[36,37]
1005	Protein	[38,39]
1095/1098	Nucleic acid (1095)	[36]
1160–1162	Carotenoid (1159)	[40]
1337/1340	Guanine, nucleic acid (1336)	[34]
1451/1452	Lipid and carbohydrates	[41,42,43]
1523–1525	Carotenoid (1523)	[40,44]
1574	Guanine, adenine of nucleic acid (1573/1575)	[34,45,46]
1660	Amide I, protein	[35,47]

## Data Availability

The original contributions presented in this study are included in the article/Supplementary Materials. Further inquiries can be directed to the corresponding authors.

## Supplementary Materials

The following supporting information can be downloaded at https://www.mdpi.com/article/10.3390/microorganisms13061333/s1, Figure S1: Identification of three growth stages of MRSA and enterotoxin-producing *S. aureus* by the established CNN model for growth stages. (A) MRSA (S6). (B) Enterotoxin-producing *S. aureus* (S63).; Table S1: Information on *S. aureus* strains used in the experiment.
