# Supplementary material for: A Pilot Study on Single-Cell Raman Spectroscopy Combined with Machine Learning for Phenotypic Characterization of Staphylococcus aureus
Source: Microorganisms. 2025 Jun 8;13(6):1333. doi: 10.3390/microorganisms13061333 (PMC12195336; doi:10.3390/microorganisms13061333)
Supplement: Supplementary file 1 [file microorganisms-13-01333-s001.zip › microorganisms-3645797-supplementary.pdf]

Table S1: Information on *S. aureus* strains used in the experiment

Strain number	Deposit number	Source of substrate	Sources of preservation
S0	ATCC 6538	The clinic	ATCC
S1	CHPC 1.8391	Food	
S2	CHPC 1.8395	Food	
S3	CHPC 1.8498	Food	
S5	CHPC 1.8507	The clinic	CHPC
S6	CHPC 1.8601	The clinic	
S16	CHPC 1.8495	The clinic	
S63	CHPC1.8462	Food	
SC1	ZD2016009	The clinic	Sichuan Province
SC2	ZD2019107	The clinic	CDC

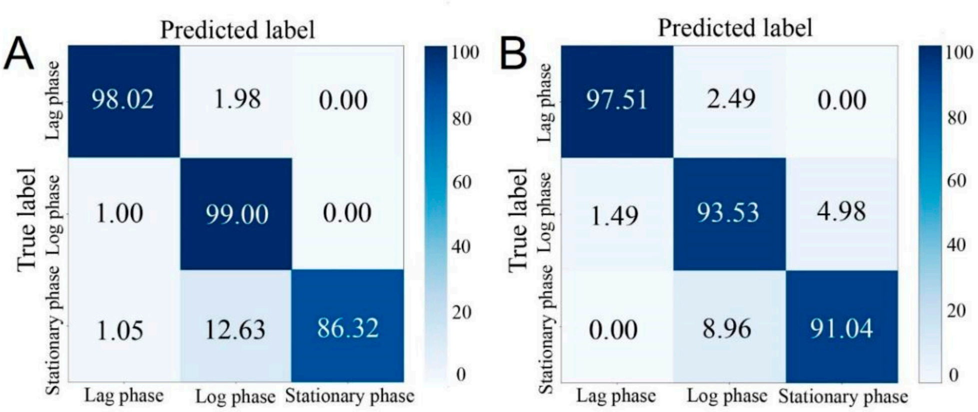

Figure S1: Identification of three growth stages of MRSA and enterotoxin-producing *S. aureus* by the established CNN model for growth stages. (A) MRSA (S6). (B) Enterotoxin-producing *S. aureus* (S63).
